# Dissociative ionization cross sections of isobutanol

**DOI:** 10.1038/s41598-025-28167-3

**Published:** 2025-11-28

**Authors:** Suriyaprasanth Shanmugasundaram, Dhanoj Gupta

**Affiliations:** Department of Physics, School of Advanced Sciences, Vellore Institute of Technology, Katpadi, Vellore, Tamil Nadu 632014 India

**Keywords:** BEB model, Isobutanol, Branching ratios, QCxMS2, Partial ionization cross sections, Chemistry, Physics

## Abstract

The electron impact partial ionization cross sections (PICS) for isobutanol were calculated using variants of the binary encounter Bethe model (BEB). The modified BEB (m-BEB) model and the mass spectrum dependent (MSD) method are used to calculate the PICS of cationic fragments of isobutanol due to electron impact. In the absence of any other data for isobutanol, the present PICS is compared with the PICS data for other isomers of butanol. We also presented the calculated electron impact mass spectrum of isobutanol, 1-butanol, and 2-butanol that is compared with the experimental mass spectrum data. Such data could be useful in the absence of any other experimental data and can be used to estimate the theoretical PICS of the molecules. Moreover, the theoretical EIMS data from quantum chemical mass spectrometry (QCxMS2) method has been used to compute the PICS of the cations.

## Introduction

Isobutanol and ethanol are important intermediates in the sugar fermentation pathway in the production of jet fuel^1^. The production of liquid fuels such as gasoline, diesel, and isooctane is derived from isobutanol through one or more chemical pathways such as blending, dehydration, oligomerization, and hydrogenation^1^. Interest in biofuels has also increased because of the risk associated with recycling and mining lithium resources worldwide.

The review of biofuels by Lopes et al.^2^ presents the studies performed until 2020 in which they have consolidated mass spectrometry, partial ionization cross sections (PICS), total cross sections (TCS), momentum transfer cross sections (MTCS), total ionization cross sections (TICS) and integrated cross sections (ICS) of alcohols such as methanol, ethanol, 1-propanol and 1-butanol. Lopes and co-workers^3–6^ have performed a complete experimental study of the electron impact mass spectrum (EIMS), appearance energies, and PICS measurements for 1-butanol and 2-butanol. The PICS for 1-butanol cations was calculated by Goswami et al.^7^ using the m-BEB method, and for 2-butanol we calculated the PICS of the cations using the MSD and m-BEB method in our previous work^8^. The results of these calculations are in reasonable agreement with the experimental PICS data. The electron impact mass spectrum (EIMS) of isobutanol was measured by Oliveira et al.^9^ at the incident energy of electron at 70 eV.

**Fig. 1:**
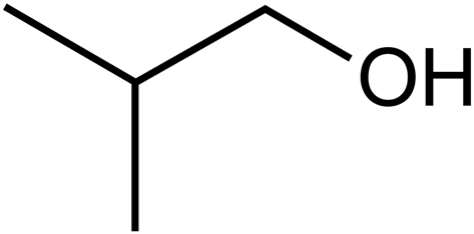
Structure of isobutanol ($\mathrm {C_4H_{10}O}$).

One of our aim in this work is to calculate the PICS for the electron impact for the isobutanol fragments by making use of the measured appearance energies and mass-spectrum data from the experiment. The MSD, m-BEB, and BEB methods in combination were used to obtain the PICS and TICS of isobutanol. The second aim is to theoretically predict the EIMS data for isobutanol, 1-butanol and 2-butanol and compare the results with the experimental measurements to check the efficacy of the quantum chemical mass spectrometry (QCxMS2)^10^ method to estimate such mass spectrum data for bigger molecules and estimate the PICS using the theoretical EIMS data. The structure of isobutanol is shown in Fig. 1. The structure of the manuscript is as follows. In section 2 we introduce the BEB model and their variants, section 3 we show the method to calculate the PICS, and, finally, section 4 contains results and discussions.

## BEB model

The BEB method^11^ calculates the electron impact ionization cross section for each orbital, and the sum of the ionization cross section for each orbital gives the total ionization cross section as shown in Eq. (1) for a target under consideration. Equation (2) gives the BEB formula for determining TICS. We use the BEB model as it is free of any fitting parameters and it is a semi-empirical model.

1 $$\begin{aligned} \sigma ^\textrm{TICS}(\mathrm{E})= & \sum _{i}^{M} \sigma _{i}~(\mathrm{E}) \end{aligned}$$

2 $$\begin{aligned} \sigma _i~(\mathrm{E})= & \alpha \left[ \frac{ \ln t_i}{2}\left( 1 - \frac{1}{t_i^{2}}\right) + \left( 1 - \frac{1}{t_{i}} \right) - k \right] \end{aligned}$$

The variable $\alpha$ houses the Burgess denominator $(t_i+u_i+1)$, which serves as an *adhoc* correction associated with the effective kinetic energy experienced by the target electron, and the $(u_i+1)$ term is acceleration due to nuclear attraction.

3 $$\begin{aligned} \alpha = \frac{4\pi a_0^2 N_i }{\left( B_i/R\right) ^2(t_i+u_i+1)} \end{aligned}$$

The term *k* holds for the electron-electron exchange effects due to the interaction of the incident electron and the ejected electron.

4 $$\begin{aligned} k = \frac{\ln t_{i}}{t_{i}+1} \end{aligned}$$

The reduced incident and orbital kinetic energy variables $t_{i},~u_{i}$ are defined as,

5 $$\begin{aligned} u_i=\frac{U_i}{B_i},~t_i=\frac{E}{B_i} \end{aligned}$$

The Bohr radius ($a_0 = 0.529\times 10^{-10}m)$, Rydberg constant (${R = 13.6~eV}$), the orbital binding energy (B), the orbital kinetic energy (U), the orbital occupation number (N), the variable (E) is the kinetic energy of the incident electron.

The BEB model has gone through several modifications to calculate the PICS of the dissociating fragments from the parent molecule; significant contributions in this area include the semi-empirical works of Hamilton et al.^12^ the theoretical approach of Irikura,^13^, the modified BEB (m-BEB) model of Goswami et al.^14^ and also the mass spectrum dependence (MSD) method of Huber et al.^15^ and Graves et al.^16^ and Wang et al.^17^ In this work, we calculate the PICS using the m-BEB formalism adopted from the works of Hamilton et al. and Goswami et al and the MSD model adopted from the works of Huber et al. and Graves et al. In our previous works,^8,18,19^ we have used the m-BEB and MSD models to calculate the PICS of various fragments that appear from 2-butanol and various other polyatomic molecules.

## Partial ionization cross sections

Oliveira et al.^9^used the HIDEN EPIC 300 quadrupole mass spectrometer to record the mass spectrum and the fragmentation curves of isobutanol. The mass spectrometer had a mass resolution of 1 amu with a mass coverage of $1 - 300$ amu and the apparatus was operated in residual gas analysis mode. The temperature was fixed to $27^\circ$C and the incident electron energy was fixed at 70 eV. The operating pressure with isobutanol in the ion source is fixed at $2\times 10^{-6}$ Torr. To measure the ionization energy (IE) and the appearance energy (AE) the ion abundance curves are fitted using the extended Wannier law.^20^ A similar experimental setup was used in the works of 1-butanol^3,4^ and 2-butanol.^5,6^ Recently the the absolute ionization cross sections of hydrocarbons have been measured using the recoil ion momentum spectrometer (RIMS) and the relative flow technique (RFT).^21,22^. Moreover, there are only few groups working on the measurement of PICS of complex molecules worldwide. On the theoretical front, there are no ab initio methods that can calculate the PICS over a wide energy range for a polyatomic molecules due to the complexity of two free electrons and a molecular ion in the exit channel.^17^ The ab-initio methods such as R-matrix^23^, Schwinger multichannel^24^ and convergent close coupling^25,26^ methods are widely used to calculate elastic and excitation cross section accurately mostly below 20 eV. Hence in such a scenario, semi-empirical methods like m-BEB^14^ and MSD^15,27^ are suitable approaches to compute the PICS of the cations of complex molecules with reasonable accuracy over a wide energy range. The m-BEB and MSD methods along with BEB model requires the mass spectrum data of the molecular target and the branching ratio of the cations dissociated from the parent molecule to compute PICS. From the electron impact mass spectrum (EIMS) data, the fragmentation yield can be determined, and the appearance energy of the cations required in the m-BEB and MSD methods is measured by fitting the ion yield curves on the Wannier threshold law in the experiments.^5^. We rely on these experimental appearance energies and mass spectrum data to compute the PICS.

For simple polyatomic molecules, the fragmentation patterns and the dissociation energy of the emerging fragment can be easily calculated. For example, for an arbitrary diatomic molecule AB, the ionization process is defined as

$$\begin{aligned} e^- + \textrm{AB} \rightarrow {\left\{ \begin{array}{ll} \textrm{A}^+ + \textrm{B} + 2e^- & \cdots \textrm{R1} \\ \textrm{A} + \textrm{B}^+ + 2e^- & \cdots \textrm{R2} \\ \textrm{AB}^+ + 2e^- & \cdots \textrm{R3} \end{array}\right. } \end{aligned}$$

In order to calculate the dissociation energy (formation energy of positive fragment) of a cation $\mathrm A^+$ we use,

6 $$\begin{aligned} \mathrm \varepsilon _i(A^+) = E(A^+) + E(B) - E(AB^+) + IP_{AB} \end{aligned}$$

similarly for cation $\mathrm B^+$

7 $$\begin{aligned} \mathrm \varepsilon _i(B^+) = E(B^+) + E(A) - E(AB^+) + IP_{AB} \end{aligned}$$

The dissociation energy of the cation is denoted by $(\varepsilon)$, E(X) denotes the total electronic energy of the ion/atom i.e. $X = A,B^+$ or $AB^+$ and $\mathrm{IP_{AB}}$ is the ionization potential of the neutral parent molecule.

For complex molecules, each fragment may contain several fragmentation pathways. Irikura^13^ used the EIMS from NIST^28^ to calculate the PICS. Hamilton et al.^12^ used the experimental branching ratios (BR) to calculate the PICS. Hamilton et al.^12^ also provided a modified approach to scale the reduced incident kinetic energy $(t_i)$ with respect to the dissociation energies ($\varepsilon$) of the respective positive ion as shown below,

8 $$\begin{aligned} t_i = \dfrac{E}{B-\varepsilon } \end{aligned}$$

using this approach they have calculated the PICS of $\hbox {NF}_x (x = 1 - 3)$. Huber et al.^15^ has also provided a solution to calculate the BR using the dissociation energies of the cation. The approach of Hamilton et al was further improved as the m-BEB model presented by Goswami et al.^14^. In this work we will use the m-BEB method and the MSD method proposed by Goswami et al.^7,14^ and Graves et al^16^. These models have been tested in our previous work for 2-butanol^8^. There are several works in the literature where the m-BEB and the MSD frameworks have been used to calculate the PICS^7,14,16,18,19,27,29,30^ and recently Lemishko et al.^31^ showed a machine learning based approach to study the fragmentation patterns of electron impact dissociation.

### Branching ratios

Typically, EIMS measurements are performed at an incident energy of 70 eV or 100 eV or at the reference energy $(\mathrm{E_r})$ used in the experimental setup that gives the relative abundances of the cations of the parent molecules from which we can define the experimental branching ratio $\Gamma _i\mathrm (E_{r})$ as follows.

9 $$\begin{aligned} \Gamma _i\mathrm{(E_{r})} = \dfrac{\textrm{R}(\textrm{E}_{\textrm{r}})}{\textrm{T}(\textrm{E}_{\textrm{r}})} \end{aligned}$$

$\mathrm R(E_{r})$ and $\mathrm T(E_{r})$ are the relative ion intensity and the total ion intensity of the cations. In the experiment, at each incident energy, the ion yields are not the same, and hence the branching ratios are dependent on the incident energy.

### MSD method

In the MSD method, the BR from the EIMS data $\mathrm \Gamma _i(\mathrm E_r)$ is scaled to reproduce the energy-dependent BR.

10 $$\begin{aligned} \Gamma _i^{\textrm{MSD}}({\textrm{E}}) = {\left\{ \begin{array}{ll} 0 & \text { if } {\textrm{E}}<\varepsilon \\ \Gamma _i({\textrm{E}}_{\textrm{r}})\left[ 1 - \left( \dfrac{\varepsilon }{E} \right) ^ z \right] & \text { if }{\textrm{E}}\ge \varepsilon \end{array}\right. } \end{aligned}$$

The control parameter *z* is set to $1.5 \pm 0.2$ by Janev and Rieter^32^ and we have fixed it as $z = 1.5$ in the present work. More details on the MSD method can be found in various sources in the literature^8,16,27^. Once we have the energy dependent branching ratio, the PICS of the cation fragments can be calculated by multiplying the TICS obtained for a parent molecule for each incident energy with the corresponding branching ratio as follow,

11 $$\begin{aligned} \sigma ^\textrm{PICS}_i\mathrm{(E)} = \Gamma _i^\textrm{MSD}(\mathrm{E}) \times \sigma _i^{\textrm{TICS}}\mathrm{(E)} \end{aligned}$$

### m-BEB

The key feature of the m-BEB model developed by Goswami et al.^7,14^ is that the dissociated cations of the parent molecule can be identified with respect to their dissociation energies ($\varepsilon$). The approach we take is by adding a correction term $(\delta )$ to the orbital binding energies of the parent molecule. The $(\delta )$ is defined as the difference between the dissociation energy of the cation and the ionization potential of the parent molecule, in short $\delta = |\text {IP} - \varepsilon |$. Such correction respectively to each of the cations alters the energy of the highest occupied molecular orbital to be the cation’s $\varepsilon$. We can assume the modified binding energy as $B'$, now we just replace the $B \rightarrow$ $B'$ in the equation. (5), the S, *k*, $u_i, ~\mathrm and~ t_i \rightarrow$, $k'$, $u_i'$ and $t_i'$, as the factor $\alpha$ holds all these values of $u_i$, and $t_i$, which then becomes $\alpha '$

12 $$\begin{aligned} \alpha '= & \frac{4\pi a_0^2 N_i }{\left( B'_i/R\right) ^2(t'_i+u'_i+1)} \end{aligned}$$

13 $$\begin{aligned} \sigma _i^{m-BEB}~(E)= & \alpha ' \left[ \frac{ \ln {t_i'}}{2}\left( 1 - \frac{1}{t_i'^{2}}\right) + \left( 1 - \frac{1}{t_{i}'} \right) - k' \right] \end{aligned}$$

After modification of the orbital binding energies to match the appearance energies of the cations, the PICS (called the unscaled m-BEB cross section) obtained for each cation is further scaled by a scaling factor $\Upsilon _i (\mathrm E_r)$, which reduces the magnitude of the cross section. This matches with the relative contribution of the cation with respect to its branching ratio from the EIMS. The scaling factor $\Upsilon _i$, is calculated from the ratio of the experimental BR $(\Gamma _i)$ and the theoretical BR $(\Gamma _i^T)$ as shown below,

14 $$\begin{aligned} \Upsilon _i\mathrm{(E_r)} = \frac{\Gamma _i(\mathrm E_r) }{\Gamma _i^{T}(\mathrm E_r)} \end{aligned}$$

In the above equation, the running indices *i* represent the cations dissociated from the parent molecule and $\mathrm{E_r}$ represents the incident kinetic energy of reference, which is 70 eV in the present case. The scaling factor in Eq. (14) is the ratio of the experimental to theoretical branching ratio at 70 eV. This scaling factor at single energy is used in m-BEB method to scale the PICS for each cation. Since experimental EIMS data is presented at only one energy of 70 eV, we could not make the scaling factor energy dependent. To calculate the theoretical BR, the ratio of the unscaled m-BEB cross section to the total ionization cross sections of the parent molecule at 70 eV is taken.

15 $$\begin{aligned} \Gamma _i^{T}(\mathrm E_r) = \frac{\sigma _i^{m-BEB}(\mathrm E_r) }{\sigma _i^{BEB}(\mathrm E_r)} \end{aligned}$$

To avoid any ambiguity in the cross sections, it is advised to know the reference energy in which the mass spectrum is measured and then do for the same energy. Once the scaling factor is obtained we can calculate the m-BEB PICS using,

16 $$\begin{aligned} \sigma ^\textrm{PICS}_i (\mathrm E) = \Upsilon _i \times \sigma ^{m-BEB}(\mathrm E) \end{aligned}$$

From Eq. (11), and Eq. (16), the electron impact partial ionization can be calculated. The cations dissociating from the parent molecule originate from one or more molecular orbitals, which could be a linear combination of molecular orbitals with a certain mixing ratio. Understanding the mixing of molecular orbitals using quantum chemistry approaches is expensive. So far, there are not enough protocols to perform such studies on orbital mixing ratios of cations. Typical coincidence experiments ($e,~e~+~$ion)^33,34^ and photoelectron spectroscopy can be used to analyze mixing ratios.

## Results and discussions

Orbital binding and kinetic energies were calculated using Gaussian-16^35^ quantum chemistry software for the optimized geometry of isobutanol. Geometry optimization was performed using density functional theory (DFT) with the $\omega \mathrm{B97XD}$ functional and the aug-cc-pVTZ basis set. The energy calculation was performed at the Hartree-Fock (HF) approximation using the same basis set. Highest occupied molecular orbital (HOMO) resulted 9.79 eV in DFT calculations, 11.94 eV in HF calculations and 13.52 eV in the QCxMS2 calculations using the GFN2-xTB level of theory.^36^ The reason for three different values with three different methods is due to inclusion of electron correlation through exchange–correlation functionals in DFT, HF approximation excludes electron correlation, and GFN2-xTB is a low-cost semi-empirical tight binding approach which provides us with an acceptable solution but not so accurate. The DFT results are in good agreement with the literature value of 10.11 eV^37,38^ as expected. Table. 1, contains the ionization potential calculated using the HF, DFT, and QCxMS2 methods for isobutanol along with the comparison of the ionization potential of its isomers from the literature^8,28,37–39^. The calculated binding energy (B) and the kinetic energies (U) of the molecular orbitals using the HF method are shown in Table 2, and are used to compute TICS of the parent molecule using the BEB model.

The TICS of the parent molecule in combination with the EIMS data can be used to compute PICS of the cations using the m-BEB and MSD methods. Branching ratios are an important factor in the calculation of the PICS. In the present study, we have used the experimental EIMS data and the appearance energies of Oliviera et al.^9^ for the calculation of branching ratio and PICS of the cations. In the absence of experimental data, theoretical approaches such as quantum chemical mass spectrometry (QCxMS2)^10^ could be used to calculate theoretical EIMS data. We have calculated the theoretical EIMS of isobutanol, 1-butanol and 2-butanol and compared with the experimental mass spectrum data.

**Table 1:** Ionization energy of isobutanol and its isomers: calculated HOMO and literature values.

Molecule	HOMO (eV)	Literature (eV)
isobutanol	9.79 (DFT), 11.94 (HF), 13.52 (GFN2-xTB)	$10.11\pm 0.07$^39^, $10.12\pm 0.04$^37^
1-butanol	–	$10.64\pm 0.07$^39^, $10.10\pm 0.05$^38^, $9.99\pm 0.05$^28^
2-butanol	9.725^8^, 11.790^8^	$9.88\pm 0.07$^39^, $9.88\pm 0.03$^37^

**Fig. 2:**
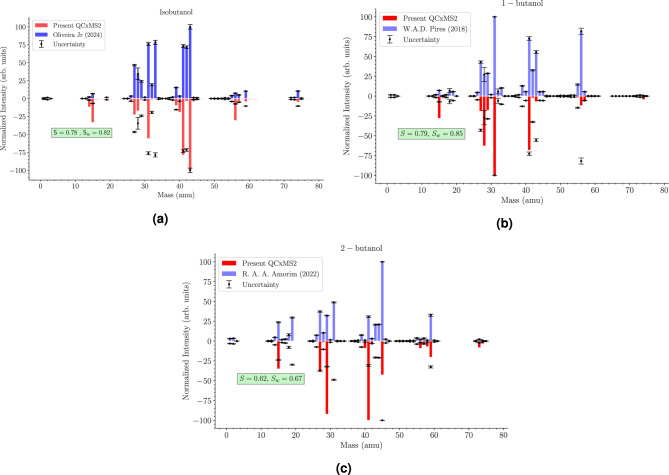
We present our electron impact mass spectrum using calculated with the QCxMS2 suite^10^ and the experimental measurements of Oliveira et al.^9^ for isobutanol in figure (**a**), W.A.D Pires et al.^3^ for 1-butanol in figure (**b**), and the measurements of R.A.A. Amorim for 2-butanol^5^ in figure (**c**) all plots contain the uncertainties and their similarity scores within. The incident kinetic energy is 70 eV in the experiment as well as the QCXMS data as well.

In Fig. 2, we have shown the comparison of the EIMS measurement of Oliveira et al.^9^ and the present calculations of EIMS using QCxMS2 for isobutanol. As 1-butanol and 2-butanol are isomers of isobutanol we have also performed the QCxMS2 calculations for them as well and compared with the existing measurements of Pires et al.^3^ and Amorim et al.^6^. A qualitative agreement between the present QCxMS2 EIMS data and the experimental data is observed with some disagreements in the magnitude of the relative abundances of the cations. However, in the absence of any other experimental/theoretical EIMS data, such an estimate of the EIMS data could be useful for targets with no available data. In QCxMS2, the EIMS quantum chemistry calculations were performed at the Grimme’s semiempirical second-generation tight-binding (GFN2) level^36^ for geometry optimizations and determination of the ionization potential using the $\mathrm \omega$B97X-3c functional from ORCA^40^ for transition state calculations. From Fig. 2, we can see that the cosine score (*S*) and weighted cosine score ($S_w$) are 0.78 and 0.83 for isobutanol. For 1-butanol the values of *S* and $S_w$ are 0.79 and 0.85 for 2-butanol the values are 0.62 and 0.67 respectively. Similar QCxMS2 calculations are also performed for several molecules^10^, which also includes 1-butanol, the QCxMS2 data calculated using (GFN2-xTB//GFN2-xTB), composite method (GFN2-xTB//$\omega$B97X-3c) and the ($\omega$B97X-3c//$\omega$B97X-3c) are compared with the NIST, and the similarity scores are 0.753, 0.750 and 0.724. This tells us that there is reasonable agreement between the EIMS measurements and the present QCxMS2 calculations. The experimental uncertainty has also been included from the EIMS measurements. The present relative intensities (RI) of some cations are shown in the table 3, together with the experimental EIMS measurements.^9^ Except for a few major cations, the relative intensities vary between the present calculations and the experimental data. The disagreement between the present calculations and the experimental measurements may be due to the inability of the present calculations in QCxMS2 to accurately model the experimental conditions. However, in the absence of any experimental data, such calculated data could be useful. The branching ratios obtained from the experimental EIMS data of Oliveira et al. and the theoretical branching ratios calculated from the QCxMS2 EIMS data, the maximum PICS of the various cations calculated using MSD and m-BEB method using the branching ratios of Oliveira et al. and QCxMS2 are presented in table 4.

**Table 2:** The orbital binding energies (B) and orbital kinetic energies (U) are presented, calculated using the RHF/aug-cc-pVTZ method, the occupation number (N) is 2 for every orbital.

MO	B (eV)	U (eV)	MO	B (eV)	U (eV)
1A	559.321	794.26	12A	17.71	29.42
2A	306.66	436.08	13A	16.52	34.27
3A	305.34	435.94	14A	15.97	31.08
4A	305.08	435.90	15A	14.74	32.32
5A	304.83	435.89	16A	14.52	41.87
6A	37.01	68.63	17A	14.36	32.12
7A	29.36	36.44	18A	12.95	38.16
8A	25.24	34.74	19A	12.84	34.74
9A	24.85	40.64	20A	12.41	36.79
10A	20.95	34.27	21A	11.94	47.64
11A	18.92	45.17			

**Fig. 3:**
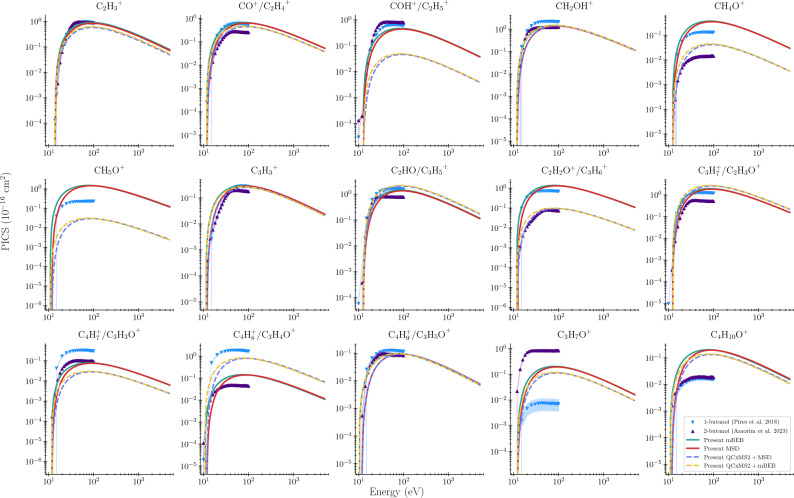
Our calculated partial ionization cross sections of isobutanol using m-BEB (solid green line) and MSD method (solid red line) are compared with the experimental measurements of 1-butanol^3^(inverted triangle) and 2-butanol^6^(upright triangle).

Figure 3, shows the comparison of the calculated PICS for isobutanol with the experimental data of 1-butanol^3^ and 2-butanol^41^ for electron impact. Since the PICS of isobutanol is unavailable in the literature, we are comparing the present PICS with the measurements of isobutanol isomers, such as 1-butanol and 2-butanol. We used Olivera et al.^9^ EIMS data in the present calculations of the branching ratio and PICS, as they have carried out the experiment and presented the relative abundances of the cationic fragments. They have compared their results with the EIMS data present in the NIST Web book^28^ and the spectral database of organic compounds (SDBS)^42^. The table 4 contains the dissociation energy of the cations measured ($\varepsilon$) by Olivera et al.^9^ and the branching ratios $(\Gamma _i)$. In the same table, we have compared the calculated electron impact maximum PICS of isobutanol with the maximum PICS of 1-butanol and 2-butanol available in the literature.^3,6–8^ As shown in our previous study on 2-butanol,^8^ the calculated PICS has a good comparison with the experimental cross sections for most of the cations. The PICS of $\mathrm {C_2H_3}^+$ agrees very well with the measurements of 1-butanol and 2-butanol; the contribution of the fragment to isobutanol, 1-butanol, and 2-butanol is 43.63, 43.0, and 37.35 percentages. A similar trend can be seen for the cation $\mathrm {CO^+/C_2H_4}^+$, where abundances are 24.05, 27.17 and 10.43 percentages for the cation in the measurements of isobutanol, 1-butanol, and 2-butanol. These trends can be observed in cations such as $\mathrm {CH_2OH}^+$ and $\mathrm {C_3H_3}^+$. For all other cations of isobutanol, the differences in the PICS are huge if we look at the cations that are common in 1-butanol and 2-butanol, this is due to the variation in relative abundances and appearance energies of the cations. The detailed cationic abundances of isobutanol, 1-butanol, and 2-butanol are shown in table 3.

It has been verified that the cation identified as base peak (with RI = 100) will have a larger PICS compared to the cross sections of other cations in their set (i.e., for $\sigma _{\max }(\mathrm {CH_5O^+})$ is $\approx 78\%$ of TICS of isobutanol), this is true for all and can be verified. All the PICS contributions of the other cations with respect to the base cation are the same as their relative abundance. These assumptions have been observed from the PICS compared in figure 3, and the relative abundances seen from the table 3.

**Table 3:** Relative abundances of cations of isobutanol compared with the reported abundances of 1-butanol and 2-butanol cations at 70 eV incident energy.

*m*/*z*	Cation	isobutanol		1-butanol		2-butanol	
		Present	Oliveira^9^	Present	W.A.D. Pires^3^	Present	R.A.A. Amorim^5^
		(QCxMS2^10^)		(QCxMS2^10^)		(QCxMS2^10^)	
27	$\text {C}_2\text {H}_3^{+}$	22.57	46.63	19.17	43.00	38.0	37.35
28	$\text {CO}^{+}/\text {C}_2\text {H}_4^{+}$	17.28	34.56	62.98	27.17	1.88	10.43
29	$\text {COH}^{+}/\text {C}_2\text {H}_5^{+}$	1.78	24.05	17.99	26.68	92.25	32.22
31	$\text {CH}_2\text {OH}^{+}$	55.76	76.28	100	100.00	0.19	48.82
32	$\text {CH}_4\text {O}^{+}$	1.62	19.28	1.25	5.94	0.00	0.57
33	$\text {CH}_5\text {O}^{+}$	1.22	78.51	0.18	10.28	0.00	0.17
39	$\text {C}_3\text {H}_3^{+}$	9.84	15.40	–	12.98	0.04	7.57
41	$\text {C}_3\text {H}_5^{+}/\text {C}_2\text {HO}^{+}$	78.87	73.24	68.3	72.78	100	30.78
42	$\text {C}_3\text {H}_6^{+}/\text {C}_2\text {H}_2\text {O}^{+}$	3.62	71.68	2.52	32.60	3.25	3.15
43	$\text {C}_3\text {H}_7^{+}/\text {C}_2\text {H}_3\text {O}^{+}$	100.00	100.00	7.0	55.62	0.05	20.76
45	$\text {C}_2\text {H}_5\text {O}^{+}$	1.03	1.16	0.31	5.68	42.6	100
55	$\text {C}_4\text {H}_7^{+}/\text {C}_3\text {H}_3\text {O}^{+}$	1.09	4.06	1.75	14.48	0.29	1.23
56	$\text {C}_4\text {H}_8^{+}/\text {C}_3\text {H}_4\text {O}^{+}$	30.56	7.48	12.18	89.93	9.29	1.81
57	$\text {C}_4\text {H}_9^{+}/\text {C}_3\text {H}_5\text {O}^{+}$	4.37	5.05	6.97	5.55	3.60	3.51
59	$\text {C}_3\text {H}_7\text {O}^{+}$	1.08	10.42	0.57	0.33	20.36	32.65
74	$\text {C}_4\text {H}_{10}\text {O}^{+}$	5.11	10.46	4.53	0.73	3.41	0.73

The $\mathrm {C_3H_7}^+$ cation ($m/z = 43$), observed in the mass spectrum, has the highest abundance. Including the base fragment $\mathrm {C_3H_7}^+ (43)$, other cations such as $\mathrm {C_2H_3}^+(27)$, $\mathrm {CH_2OH}^+(31)$, $\mathrm {CH_5O}^+(33)$, $\mathrm {C_2HO}^+/\mathrm {C_3H_5}^+(41)$, $\mathrm {C_2H_2O}^+/\mathrm {C_3H_6}^+(42)$ contribute to the abundance 50 % in the mass spectrum. This also implies summing up their PICS yields 50 % of the TICS. In table 4, we have shown the comparison of the maximum PICS of the 15 cationic fragments. All 15 of these cations have also been detected in 1-butanol and 2-butanols, and they have a different relative intensity. Most of the 2-butanol cations in this set except $\mathrm {CH_5O} ^+(33)$ have experimental PICS data. Since $\mathrm {CH_5O}^+$ only had an abundance of 1.16%, it must be difficult to measure its PICS in an experiment for isobutanol. Studies on 1-butanol and 2-butanol^7,8^ show that PICS agrees well with experimental measurements where the incident energies are greater than 35 eV. The difference in cross-sectional peak positions of cations observed in the present calculations and in the experiment is due to the dependence of the cross section on the branching ratio and the appearance energies of the cations. Similar differences were also observed between the theoretical and experimental PICS data of some cations of 2-butanol as presented in table 4. More investigation are necessary to validate the current set of data. The general trend of the cross section for electron impact appears to follow a similar trend as seen by Graves^27^. In the same figure, we have presented the PICS calculated using the theoretical EIMS and BR obtained from the QCxMS2 using the MSD and m-BEB models. Here we have used the appearance energies of Oliveira et al.^9^ for the cations. The dashed lines in the figure 3 represents the QCxMS2$+$MSD (blue) and QCxMS2$+$m-BEB (yellow). Unsurprisingly, cations with similar BR such as $\mathrm{CH_2OH^+}$,$\mathrm {C_3H_3^+}$,$\mathrm{C_4H_9^+/C_3H_5O^+}$ have similar PICS maxima. The cations $\hbox {C}_2\hbox {H}_3^{+}$,$\hbox {CO}^{+}$/$\hbox {C}_2\hbox {H}_4^{+}$,$\hbox {C}_2\hbox {HO}^{+}$/$\hbox {C}_3\hbox {H}_5^{+}$,$\hbox {C}_3\hbox {H}_7^{+}$/$\hbox {C}_2\hbox {H}_3\hbox {O}^{+}$ have the slightly lower PICS compared to the experimental results. The PICS of some cations show a large differences due to the differences in the theoretical EIMS data calculated using QCxMS2 and the experimental EIMS data of Oliveira et al.^9^. However, the PICS results obtained from QCxMS2 are encouraging at least for some prominent fragments and will be explored more in future. Also it is worth noting that the results obtained from both MSD and m-BEB methods are similar in shape and magnitude with a small variation slightly above the threshold energies for all the cations. The variation may be due to the branching ratio and scaling factor which is calculated at one energy in the m-BEB model but in the MSD method the branching ratio is made energy dependent that scales the TICS at each corresponding energies.

**Fig. 4:**
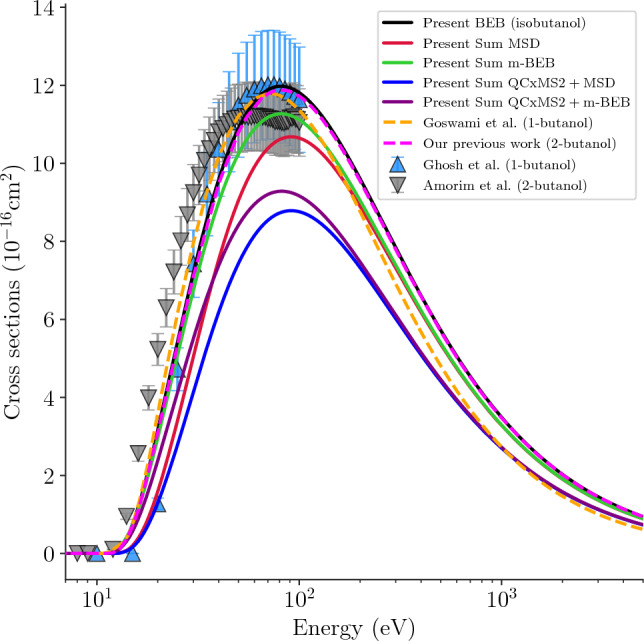
Our BEB TICS of isobutanol compared with the literature 1-butanol and 2-butanol. Present BEB TICS isobutanol (solid black line), sum of MSD PICS (solid red line), sum of m-BEB PICS (solid green line), sum of QCxMS2$+$MSD PICS (solid blue line), sum of QCxMS2$+$m-BEB PICS (solid purple line), BEB TICS of 1-butanol by Goswami et al.^7^ (dashed orange line), BEB TICS of 2-butanol from our previous work^8^ (dashed magenta line), measured TICS of 1-butanol by Ghosh et al.^4^ (upright blue triangle), measured TICS of 2-butanol by Amorim et al.^6^ (inverted gray triangle).

In Fig. 4, we have compared the TICS of isobutanol with their isomers for electron impact along with the summation of PICS calculated using MSD, m-BEB, QCxMS2$+$MSD, QCxMS$+$m-BEB. The plot shows that the magnitudes of the present TICS of isobutanol are slightly higher than the BEB TICS of 1-butanol^7^ and 2-butanol.^6,8^ The present sum of PICS calculated using MSD and m-BEB from the experimental EIMS data fall within the experimental uncertainty of 1-butanol and 2-butanol. But the sum of PICS calculated using QCxMS2$+$MSD/m-BEB falls short as expected as the EIMS data is not predicted accurately for many cations compared to experiment. It is well know that the TICS calculated using the BEB model with HF parameters falls within the experimental uncertainty of $10\% - 15\%$^43,44^. The same TICS with the uncertainty of $10\% - 15\%$ is scaled using the branching ratios to calculate the PICS of the cations. Hence the uncertainty in the PICS data increases by more than $15\%$. The factors that can increase the uncertainty in the PICS are the EIMS data, the branching ratio and the appearance energies. Hence the accurate measurement of these quantities can help in predicting the accurate PICS. In the present case we can see in the TICS comparison the sum of QCxMS2$+$MSD, QCxMS$+$m-BEB are lower than the BEB TICS clearly showing the uncertainty that comes from the theoretical EIMS data that does not fully match with the experimental data.

**Table 4:** The branching ratios and electron impact partial cross sections of isobutanol are compared with the literature data of 1-butanol and 2-butanol. $(\blacklozenge )$ Oliveira et al.^9^ experiment, $(\dagger )$ Pires et al.^3^ experiment, $(\bigstar )$ Goswami et al.^7^ m-BEB calculation, ${(\ddagger )}$ Amorim et al.^6^ experiment, $(\blacktriangle )$ MSD and m-BEB data of 2-butanol from our previous work.^8^.

		isobutanol			$\mathrm \sigma ^{max} (E) ~~[\mathrm 10^{-16}cm^2, eV]$								
*m*/*z*	Cations	$\varepsilon$ (eV) ^9^	$\Gamma _i~@70$ eV		Present isobutanol				1-butanol		2-butanol		
			$\hbox {Exp.}^{\blacklozenge }$ ^9^	Present	MSD	m-BEB	QCxMS2		$\hbox {Exp.}^{\dagger }$ ^3^	$\hbox {m-BEB}^{\bigstar }$ ^7^	$\hbox {Exp.}^{\ddagger }$ ^6^	$\hbox {MSD}^\blacktriangle$ ^8^	$\hbox {m-BEB}^\blacktriangle$ ^8^
							MSD	m-BEB					
27	$\mathrm{C_2H_3^+}$	13.99	0.0741	0.0518	0.852(93.5)	0.925(90.0)	0.581(93.5)	0.630(90.0)	0.965(70.0)	0.946(80.0)	0.964(48.0)	0.338(90.0)	0.366(90.0)
28	$\mathrm{CO^+/C_2H_4^+}$	11.64	0.0549	0.0397	0.641(90.5)	0.674(80.0)	0.452(90.5)	0.475(80.0)	0.616(60.0)	0.596(75.0)	0.279(50.0)	0.287(90.0)	0.301(80.0)
29	$\mathrm{COH^+/C_2H_5^+}$	12.6	0.0382	0.0041	0.444(92.0)	0.472(84.0)	0.046(92.0)	0.049(84.0)	0.644(70.0)	0.629(75.0)	0.834(44.0)	0.332(90.0)	0.359(90.0)
31	$\mathrm{CH_2OH^+}$	11.79	0.1212	0.1281	1.414(91.0)	1.488(80.5)	1.457(91.0)	1.533(80.5)	2.245(65.0)	2.306(70.0)	1.271(90.0)	0.317(90.0)	0.338(85.0)
32	$\mathrm{CH_4O^+}$	12.19	0.0306	0.0037	0.356(91.5)	0.377(82.0)	0.042(91.5)	0.044(82.0)	0.134(75.0)	0.130(70.0)	0.015(90.0)	0.307(90.0)	0.326(85.0)
33	$\mathrm{CH_5O^+}$	10.96	0.1247	0.0026	1.463(90.0)	1.526(77.0)	0.030(90.0)	0.031(77.0)	0.235(95.0)	0.225(70.0)	–	–	–
39	$\mathrm{C_3H_3^+}$	11.17	0.0244	0.0226	0.287(90.0)	0.300(78.0)	0.258(90.0)	0.270(78.0)	0.290(75.0)	0.282(70.0)	0.198(48.0)	0.261(100.0)	0.272(75.0)
41	$\mathrm{C_3H_5^+/C_2HO^+}$	12.84	0.1164	0.1812	1.349(92.0)	1.439(85.0)	2.047(92.0)	2.183(85.0)	1.633(70.0)	1.593(70.0)	0.801(34.0)	0.304(90.0)	0.322(85.0)
42	$\mathrm{C_3H_6^+/C_2H_2O^+}$	11.6	0.1139	0.0083	1.330(90.0)	1.397(79.5)	0.095(90.5)	0.099(79.5)	0.733(65.0)	0.714(70.0)	0.081(56.0)	0.265(90.0)	0.275(75.0)
43	$\mathrm{C_3H_7^+/C_2H_3O^+}$	12.11	0.1589	0.2297	1.850(91.0)	1.954(82.0)	2.606(91.0)	2.754(82.0)	1.248(70.0)	1.218(70.0)	0.566(40.0)	0.291(90.0)	0.306(80.0)
55	$\mathrm{C_4H_7^+/C_3H_3O^+}$	11.58	0.0064	0.0025	0.075(90.5)	0.079(79.5)	0.029(90.5)	0.030(79.5)	0.333(60.0)	0.321(70.0)	0.102(46.0)	0.304(90.0)	0.322(85.0)
56	$\mathrm{C_4H_8^+/C_3H_4O^+}$	10.5	0.0118	0.0702	0.140(89.5)	0.145(75.0)	0.805(89.5)	0.836(75.0)	1.873(55.0)	1.794(70.0)	0.048(42.0)	0.237(90.0)	0.243(70.0)
57	$\mathrm{C_4H_9^+/C_3H_5O^+}$	10.07	0.0080	0.0082	0.094(89.0)	0.098(73.0)	0.094(89.0)	0.098(73.0)	0.127(55.0)	0.122(70.0)	0.097(34.0)	0.281(90.0)	0.295(80.0)
59	$\mathrm{C_3H_7O^+}$	12.06	0.0165	0.0100	0.193(91.0)	0.204(81.5)	0.114(91.0)	0.120(81.5)	0.008(45.0)	0.007(70.0)	0.840(90.0)	0.272(100.0)	0.284(80.0)
74	$\mathrm{C_4H_{10}O^+}$	10.61	0.0166	0.0117	0.196(89.5)	0.204(75.5)	0.134(89.5)	0.139(75.5)	0.017(60.0)	0.016(70.0)	0.020(48.0)	0.250(90.0)	0.257(75.0)

## Conclusion

The electron impact PICS and TICS of isobutanol have been calculated. It is found to be more stable in the $\mathrm {C_1-C_4}$ alcohol set^2^. It has also been shown to be much more stable than their isomers, such as 1-butanol, 2-butanol, or *n*-butanol. Hence, this could be used in a study that seeks a viable alternative to current fossil fuels^9^. The TICS results showed reasonable agreement with the literature data. The PICS resulting from electron impact have been calculated and presented for the first time in the literature. The QCxMS2 program has been tested to calculate the electron impact mass spectrum of isobutanol, 1-butanol and 2-butanol and a reasonable agreement is found with the experimental data, looking at the similarity scores. The PICS calculated using QCxMS2$+$MSD/m-BEB for a few cations matches with the PICS calculated using the BR of Oliveira et al.^9^ QCxMS2 calculations are important when there is no experimental investigation of the EIMS data. In the future, our work will be to independently investigate the EIMS data and use those in the present m-BEB and MSD models to estimate the PICS of some bigger biomolecules where there are no experimental results.

## Supplementary Information

Supplementary Information 1.

Supplementary Information 2.

Supplementary Information 3.

Supplementary Information 4.

Supplementary Information 5.

## Data Availability

Data sets generated during the current study are available in the form of electronic supplementary material.
